# Heart Rate Variability for Outcome Prediction in Intracerebral and Subarachnoid Hemorrhage: A Systematic Review

**DOI:** 10.3390/jcm12134355

**Published:** 2023-06-28

**Authors:** Luca Marino, Rafael Badenes, Federico Bilotta

**Affiliations:** 1 Department of Mechanical and Aerospace Engineering, “Sapienza” University of Rome, 00184 Rome, Italy; 2 Department of Anesthesiology and Surgical-Trauma Intensive Care, Hospital Clínic Universitari de Vacia, University of Valencia, 46010 Valencia, Spain; 3 Department of Anesthesiology, Critical Care and Pain Medicine, Policlinico Umberto I, “Sapienza” University of Rome, 00185 Rome, Italy; federico.bilotta@uniroma1.it

**Keywords:** heart rate variability, intracerebral hemorrhage, subarachnoid hemorrhage, outcome prediction

## Abstract

This systematic review presents clinical evidence on the association of heart rate variability with outcome prediction in intracerebral and subarachnoid hemorrhages. The literature search led to the retrieval of 19 significant studies. Outcome prediction included functional outcome, cardiovascular complications, secondary brain injury, and mortality. Various aspects of heart rate recording and analysis, based on linear time and frequency domains and a non-linear entropy approach, are reviewed. Heart rate variability was consistently associated with poor functional outcome and mortality, while controversial results were found regarding the association between heart rate variability and secondary brain injury and cardiovascular complications.

## 1. Introduction

Heart rate variability (HRV) is a biomarker extracted from continuous ECG analysis and used as a clinical severity indicator in several cardiac and non-cardiac diseases, including heart failure and acute myocardial infarction; sepsis and multiple organ dysfunction; renal disease; respiratory infection; metabolic impairment; as well as in patients with traumatic brain injury and some neurological disorders [1,2,3,4,5,6,7,8]. Recently, a systematic review addressed the role of HRV in predicting the course of patients presenting with ischemic and hemorrhagic strokes and reported that it might be a promising predictor of outcome and complications [9].

Intracerebral hemorrhage (ICH) and subarachnoid hemorrhage (SAH) are devastating diseases associated with high morbidity and mortality [10,11]. In patients with ICH, mortality can reach 50%, and functional outcome impairment complicates 32.8–42.4% at 6 months and 16.7–24.6 at 12 months [12]. Almost 35% of patients with SAH die within 3 months, and more than 50% of survivors have a permanent neurological deficit and functional impairment [13]. Several predictive scores have been proposed to anticipate functional outcome and mortality in patients presenting with ICH/SAH [14]. Most of these scores are based on initial neurological conditions, but there is no consensus on possible relevant variables extracted by the clinical course or on the optimal timing for the assessment [15].

The HRV analysis has been tested as a possible tool to assess the prognosis of patients presenting with ICH or SAH, but there is no conclusive evidence on the accuracy and value of this approach in the prediction of functional outcome, cardiovascular complications, secondary brain injury, and mortality of these patients or its association with clinical severity.

The aim of this systematic review is to focus the available clinical evidence on the possible association between HRV increase or decrease with the considered endpoints in patients with ICH/SAH.

## 2. Methods

The present systematic review has been carried out according to the Preferred Reporting and Items for Systematic Review and Meta-Analyses (PRISMA). The analysis has been recorded in the PROSPERO registry database for systematic reviews (N. CRD 42022336996, 12 June 2022).

Articles related to the predictive value of HRV in patients presenting with ICH/SAH were searched on PubMed and Google Scholar using the following keywords: “Heart rate variability”, “Heart rate variation”, “subarachnoid hemorrhage”, “intraparenchymal hemorrhage”, “cerebrovascular diseases”, and “outcome prediction”. The screening was extended to the references of the selected studies and considered documents published before July 2022.

Studies conducted as prospective randomized controlled trials (RCT) as well as prospective or retrospective observational studies, published as full papers in English in peer-reviewed journals, with ruled participants aged 18+ years, were considered suitable for the present systematic review. Exclusion criteria included preclinical and nonexperimental studies (e.g., meta-analyses, comments/letter, abstract, case reports, conference abstracts). The disagreement over eligibility was resolved through open discussion.

The following information was extracted using a standardized data extraction form: type of study, number of recruited patients, admission diagnosis (ICH, SAH), characteristics of HRV recording and analysis, and follow-up clinically recorded variables.

The HRV recording and analysis methodologies varied among the selected studies, and a description of the details is reported hereafter. Differences in tested domains included an analysis of time-domain, and frequency-domain, and entropy approaches were based on the sequence of intervals between successive R peaks of QRS complexes of the ECG [16,17], Table 1. The time-domain analysis is accomplished with different statistical measures: standard deviation of RR intervals (SDNN); normal-to-normal deviation of intervals measured between consecutive sinus beats; root-mean-square of successive beat-to-beat differences (RMSSD); mean value of HR with its standard deviation (HR-SD); and heart rate average real variability (HR-ARV). The frequency domain analysis is based on the decomposition of the signal into its frequency components and the evaluation of the relative contribution, namely the power spectral density (PSD). Statistical measures were the low and high frequency (LF, HF) components of the PSD and the ratio LF/HF as a measure of sympathovagal balance. The entropy approach was based on the analysis of heart rate fluctuations at multiple time scales. The statistical measure was the HRV complexity index, namely the richness of fluctuations in each scale analyzed [18].

## 3. Results

### 3.1. Study Characteristics

**Figure 1:**
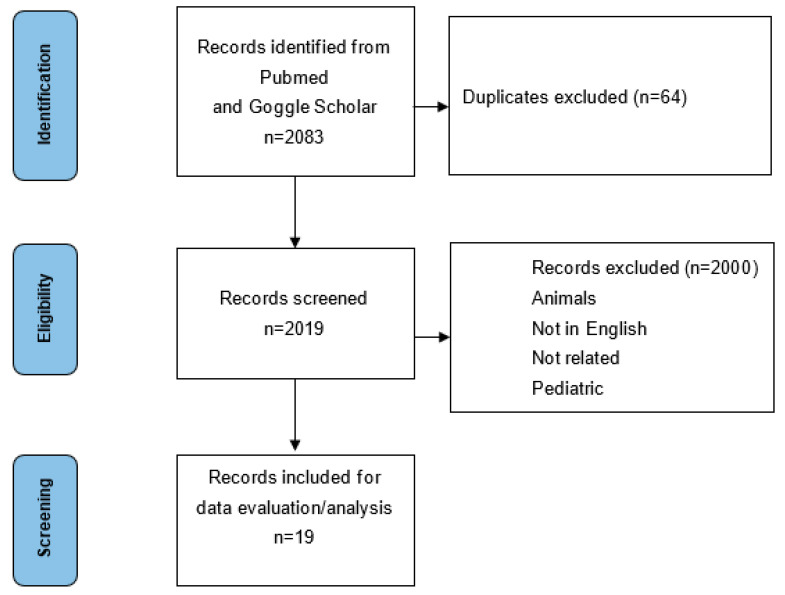
Prisma Diagram.

The literature analysis yielded a total of 2000 studies, out of these 19 were selected as appropriate for the present systematic review (Figure 1). All the 19 studies are observational, 15 prospective, and 4 retrospective (Table 2). Recorded clinical variables included the functional outcome, cardiovascular complications (neurocardiogenic damage and neurogenic pulmonary edema), secondary brain injury (delayed cerebral ischemia, inflammation, fever), and mortality. Related data are presented according to the number of studied patients.

### 3.2. Recording and Analysis of HRV

Various aspects of HRV recording (beginning, duration, and sampling frequency) and of the analysis (time, frequency domains, and entropy evaluation) differentiate the studies selected for the present systematic review (Table 2). The beginning of HRV recording varied greatly and coincided with the time of the diagnosis [24] or started within 48 h [30] after hospital/ICU admission. In one study the beginning of HRV was not specified [37]. The duration of monitoring ranged from 10 s to 21 days and included a sampling frequency that ranged from 200 Hz to 4096 Hz. In five studies, the sampling frequency was not reported [19,20,22,26,29]. In eight studies, details on the profile of time domain were given [19,20,22,24,30,32,35,37]. In 15 studies, the frequency domain approach was adopted [23,25,26,27,28,29,30,31,32,33,34,35,36,37]. In two studies, a non-linear analysis based on the multiscale entropy, was adopted in [21,36].

### 3.3. Functional Outcome

The relationship between HRV and functional outcome was analyzed in five studies [19,20,21,22,23] that included a total of 1497 patients: in four studies (three prospective and one retrospective), 1152 cases were selectively recruited with ICH [19,21,22,23] and one retrospective study recruited 345 patients with SAH [20] (Table 3). In all studies, functional outcome was reported as the primary endpoint, and the association of HRV was evaluated as an odd ratio (OR). Two scales were used to score the follow-up functional status: the modified Rankin Scale was consistently recorded at 90 days/3 months follow-up in the four studies that recruited ICH patients [19,21,22,23]; the Glasgow Outcome Scale was recorded at hospital discharge in the study that recruited SAH patients [20]. In order to detect the relationship between HRV and functional outcome, all possible domains of analysis (time, frequency, and entropy) were tested. Analysis of time domains included: HR-ARV, mean HR, and HR-SD [19,20,22]. Frequency domain analysis included LF, HF, and LF/HF ratio [21,23], and normalized PSD (for HF and LF) [23]. The entropy complexity index was evaluated in one study [21]. According to the recorded results, a higher HRV is consistently associated with poor functional outcome both in ICH and SAH patients with an OR predictive value ranging between 1.14 and 1.31 (Table 3). Accordingly, a higher HRV complexity is associated with a good functional outcome (modified Rankin Scale < 3): OR 1.09 [21].

### 3.4. Cardiovascular Complications

Cardiovascular complications, neurocardiogenic injury (NCI), and neurogenic pulmonary edema (NPE) were analyzed in five prospective observational studies that reported data from a total of 674 SAH patients, Table 4. In three studies on 396 patients with NCI, cardiovascular complications were diagnosed in 17.8% to 93% of patients [24,26,28], and in two studies on 278 patients with NPE, complications were diagnosed in 13% to 14.5% [25,27]. The occurrence of NCI was diagnosed in one study as echocardiographic abnormalities without ECG changes [24] and in two studies as ECG abnormalities [26,28]. The occurrence of NPE was diagnosed based on a chest radiograph and clinical findings [25,27]. The follow-up period ranged between 24 h and 60 days. Analysis of time domains included SDNN, RMSSD, mean HR, and HR-SD [24]. Frequency domain analysis included LF, HF, and LF/HF ratios and normalized PSD (for HF and LF) [24,25,26,27,28]. The relationship between HRV and NCI-NPE is associated with controversial evidence: a higher LF/HF was reported in two studies [24,27], a lower LH/HF in two others [25,26], and one study reported inconclusive results [28].

### 3.5. Secondary Brain Injuries

Secondary brain injury was reported in five studies on 608 patients as delayed cerebral ischemia (DCI), inflammatory cytokines production, and fever occurrence (Table 5). Delayed cerebral ischemia was registered in three studies—two prospective and one retrospective—on 346 patients with a diagnosis of SAH [30,31,32]. A prospective study on 14 patients (six ICH and eight SAH) analyzed inflammatory cytokines production [33], and a prospective study on 248 patients with ICH considered fever occurrence [29]. DCI was recorded in 14% to 21% patients, fever occurrence in 47%, and inflammatory cytokines production in 50%. The follow-up period ranged between 4 days and 1 year. Analysis of time domains included SDNN, RMSSD, mean HR, and HR-SD [29,32]. Frequency domain analysis included LF, HF, and LF/HF ratios and their normalized PSD values [30,32,33]. The association of low HRV and DCI development was recorded in one study [31] and was not predictive in two studies [30,32]. Lower HRV was associated with greater odds of fever occurrence in one study [29]. Higher normalized HF power and lower LF/HF were associated with inflammatory cytokines production in one study [33].

### 3.6. Mortality

Mortality was registered in six studies—five prospective and one retrospective—on 345 patients (298 with diagnosis of SAH and 47 of ICH) [27,32,34,35,36,37], Table 6. Mortality was recorded in 16% to 54% patients. The follow-up period ranged between 7 days and 1 year. Analysis of time domains included SDNN, RMSSD, mean HR, and HR-SD [32,37]. Frequency domain analysis included LF, HF, and LF/HF ratios and their normalized PSD values [27,32,34,35,36,37]. A lower HRV was associated with a poor outcome in five studies (four on 266 with SAH and one on 47 patients with ICH) [27,32,34,35,36]. In a study on 24 patients with SAH, HRV was not predictive of mortality [37].

## 4. Discussion

This systematic review reports original clinical evidence on the relationship between HRV and functional outcome, cardiovascular complications, secondary brain injury, and mortality in patients with ICH/SAH. HRV recording and analysis were carried out with various methodological approaches, based on linear time and frequency domains and nonlinear methods. HRV was consistently associated with poor functional outcomes and mortality. Controversial results were found regarding the association of HRV with secondary brain injury and cardiovascular complications.

The HRV is principally controlled by the dynamic relationship of the sympathetic and parasympathetic nervous systems with the autonomic nervous system [38]. The preganglionic cholinergic sympathetic neurons of the heart are located in the intermediolateral cell column of the upper thoracic spinal cord and make synapses in the cervicothoracic stellate ganglia [39]. The noradrenergic postganglionic sympathetic neurons innervate the cardiac conduction system, atria, and ventricles, and, through a signaling pathway linked to the β_1_ receptors of the myocytes, improve the stress related response. The parasympathetic system, through the vagus nuclei, innervates, and, in particular, the atrial conduction system [3]. Hence, a decrease in HRV corresponds to the activation of sympathetic system (i.e., the response to stressors), whereas an increase is observed for the activation of the parasympathetic system [40] (i.e., the rest-and-digest response).

Some arguments support the role of the parasympathetic system dysfunction after ICH/SAH to induce successive fever and inflammatory cytokines expression, which in turn contributes to worsen the outcome. The associated elevated levels of excitatory amino acids, free radicals, lactic acid, blood–brain barrier breakdown, and impaired enzymatic function can be related to secondary brain injury through cerebral edema, a reduction in cerebral perfusion pressure, and DCI [41,42,43]. The clinical impact is not fully understood, and it is still matter of ongoing research.

Evidence of the predictive role of the imbalance of the autonomic nervous system, witnessed by HRV, is reported in several pathological conditions, including sepsis, congestive heart failure, ischemic cardiomyopathy, respiratory diseases, spinal cord injury, stroke, epilepsy, and neuropsychiatric and neurodegenerative diseases [44,45,46,47,48]. As an example, the role of HRV analysis has been deeply investigated in sepsis [44] and, among the HRV parameters verified to predict risk of death, those based both on time domain and frequency domain were revealed to be reduced in non-surviving septic patients. In particular, SDNN was found to be independently associated with mortality in sepsis and emerged as a useful HRV parameter to predict sepsis outcome. A further noticeable example is given by the role of HRV as a risk marker for the prediction of cardiovascular mortality in myocardial infarction and heart failure [1]. Depressed HRV, in particular computed by SDNN values, was associated with increased mortality both in post myocardial infarction and chronic heart failure patients. A recent systematic review focused on the association of HRV with acute stroke by considering both hemorrhagic and ischemic etiologies [9]. In that systematic review the authors highlight the HRV associations with stroke complications (mortality and stroke recurrence, infections, post-stroke depression, neurocardiogenic injury, delirium, and atrial fibrillation) and confirmed the potential role of HRV as a promising biomarker for outcome and complications prediction. Data presented in the present systematic review confirm and extend the evidence reported on HRV as a prognostic predictor in patients with stroke.

The present systematic review reports on two forms of hemorrhagic acute brain injury: ICH and SAH. These events recognize different pathophysiological mechanisms: in ICH, the bleeding is into brain parenchyma, while SAH is bleeding into the subarachnoid space [49]. Furthermore, the ICH and SAH differs in epidemiological characteristics. Overall, incidences evaluated in the extensive range of years 1980–2020—for both western and eastern countries—is 29.9 per 100,000 person-years (95% CI: 26.5–33.3) for ICH compared to 7.9 (95% CI, 6.9–9.0) per 100,000 person-years for SAH. Even if these data are averaged to the last 40 years, recent analyses restricted to the last 10 years confirm the remarkable difference for the two possible hemorrhages [50,51]. Early complications can be different between SAH and ICH. SAH complications include rebleeding, acute hydrocephalus, acute ischemic lesions, non-neurological complications (ECG repolarization alterations, Tako-Tsubo syndrome), and vasospasm [52]. Early complications for ICH include hematoma expansion, perihematomal edema, intraventricular extension of hemorrhage with hydrocephalus, seizures, venous thromboembolic events, hyperglycemia, increased blood pressure, fever, and infections [53]. Even if the two conditions differ in their anatomical locations, epidemiological features, and early complications, they share some risk factors, the most important of which being hypertension, complications, functional outcomes, and mortality [54,55]. These conditions are both associated with great autonomic disfunctions, which motivated the choice of presenting together the relationship between HRV and clinical course [56].

The subheadings of the Section 3 were designed to summarize primary and secondary endpoints as presented in the original studies and include functional outcome, secondary brain injury, cardiovascular complications, and mortality.

Of note, the studies selected for the present systematic review have a non- homogeneous approach to evaluating the HRV parameters. The Task Force of the European Society of Cardiology [16] suggested to adopt both time and frequency domains to analyze HRV characteristics to avoid controversial clinical interpretations. Non-linear methods, based on entropy analysis, are a promising measure of HRV, but controversies regarding clinical interpretation do not currently make this approach the “gold standard” to analyze HRV.

There are several possible limitations to the presented evidence. The first is the high heterogeneity of the methodological approaches used among the studies that qualified for the present systematic review, including the data recording and analysis and the types and timing of follow-up, while the second is the lack of an ultimate indication on the relationship and predictive value of HRV in patients presenting with ICH or SAH. A further possible limitation of this systematic review is the methodology, which is based on a literature search limited to two databases (PubMed and Google Scholar). In any case, the risk of skipping important information is limited due to the comprehensive features of the databases. Another limitation is the restricted number of keywords chosen to launch the literature search, which might have missed the examination of studies potentially related to the present analysis. The retrieved data did not allow a meta-analysis due to the limited number of studies on many issues. Moreover, the included studies present a great variance in terms of their design (prospective and retrospective) and the underlying pathology of the study population (SAH or ICH), which cannot be incorporated in a single analysis. Study endpoints also present considerable variance in terms of the time of assessment.

In conclusion, the HRV seems to have promising characteristics in the risk stratification of functional outcome, cardiovascular complications, secondary brain injury, and mortality. In all the studies selected for the present systematic review, the HRV analysis was accomplished offline after recording. Future studies should be designed with an appropriate methodological approach and include an early beginning of HRV monitoring starting from patients’ hospital admission, as well as a standardized analysis of the related characteristics, such as time and frequency domains, which should be extracted in a timely manner in order to test the accuracy of risk stratification. HRV online monitoring might become a useful tool to predict the clinical course of patients with acute brain injury and could be implemented as “routine” support in clinical practice.

## Figures and Tables

**Table 1:** Methodological aspects of heart rate variability analysis (HRV).

Time-Domain Measures of HRV			
Variable Symbol	Units	Description and Method to Calculate	Comments
SDNN	ms	Normal-to-normal (NN) standard deviations. Measured between consecutive QRS complex sinus beats.	Returns all the cyclic components responsible for variability in the period of recording. Dependent on length of recording period. Accurate when recorded on 24 h period.
RMSSD	ms	Root mean square of successive di differences between normal heartbeats. Measured as square root of the mean of the sum of the squares of differences between adjacent NN intervals.	Meaningful on short time recording. Reflects the beat-to-beat variance in heart rate, primary time-domain measure to estimate the vagally mediated changes existing in HRV
HR-SD	bpm	Standard deviation of successive heart rate values.	Analogous to SDNN. Not standard measure.
HR-ARV	bpm	Heart rate average real variability. Measured as absolute difference between consecutive values of heart rate	Not standard measure.
		Frequency-domain measures of HRV	
VLF	ms2	Very low frequency range of power spectral density. Measured by Fast Fourier Transform of the time domain signal and filtered to very low frequencies (<0.04 Hz).	Associated to arrhythmia, But physiological interpretation not well defined.
LF	ms2	Low frequency range of power spectral density. Measured by Fast Fourier Transform of the time domain signal and filtered to low frequencies (0.04–0.15 Hz)	Associated to both sympathetic and parasympathetic activities.
HF	ms2	High frequency range of power spectral density. Measured by Fast Fourier Transform of the time domain signal and filtered to high frequencies (0.15–0.4 Hz)	Associated mainly to parasympathetic activity.
LF/HF		Ratio of LF [ms2]/HF [ms2]	Interpretation dependent on length of recording period and measuring conditions. In a 24 h recording period, low LF/HF ratio measures parasympathetic dominance, while high LF/HF ratio implies sympathetic dominance
LF%	n.u.	100 × LF/(Total power − VLF)	More sensible to the behavior of the two branches of the auto-nomic nervous system.
HF%	n.u.	100 × HF/(Total power − VLF)	
		Non-linear measures of HRV	
Entropy		Measure of time series complexity at multiple scales (MSE).	Multiple scale entropy measures the richness of fluctuations in each scale analyzed but the interpretation is difficult.

**Table 2:** Description of characteristics of included studies.

First Author Year Reference	Number of Patients, Type of Patients, Study Design	Endpoints	Start of Monitoring	Monitoring Time	Domain Studied HRV Measures	Sampling Frequency	Follow Up Time
Miwa et al., 2021 [19]	*n* = 994, ICH, retrospective	Functional outcome	<2 h	24 h	Time HR-SD, HR-ARV SDNN, RMSSD	N.A.	90 d
Cai et al., 2018 [20]	*n* = 345, SAH, Retrospective	Functional outcomes	<24 h	24 h	Time HR-SD	N.A.	Discharge
Chen et al., 2018 [21]	*n* = 93, ICH, Prospective	Functional outcome	<24 h	1 h	Time, Frequency, MSE SDNN, RMSSD, LF, HF, LF/HF Entropy	512 Hz	3 months
Rass et al., 2021 [22]	*n* = 88, ICH, prospective	Functional outcome	<24 h	8 d	Time HR-SD	N.A.	3 months
Szabo et al., 2018 [23]	*n* = 47, ICH, prospective	Functional outcome Mortality	<24 h	10 m	Frequency LF, HF, LF/HF	200 Hz	90 d
Megjhani et al., 2020 [24]	*n* = 326, SAH, prospective	Neurocardiogenic injury	Admission	48 h	Time, Frequency MNN, SDNN, RMSSD LF, HF, LF/HF	240 Hz	48 h
Chen et al., 2016 [25]	*n*= 248, SAH, Prospective	NPE	At diagnosis	10 min	Frequency LF, HF, LF/HF	1000 Hz	24 h
Kawahara et al. 2003 [26]	*n* = 43, SAH, Prospective	Neurocardiogenic injury	Admission	Series of 24 h	Frequency LF, HF, LF/HF	N.A.	30 d or more
Su et al., 2009 [27]	*n* = 30, SAH, prospective	mortality DCI NPE	<48 h	3 d	Frequency LF, HF, LF/HF	500 Hz	1 w
Svigelj et al. 1996 [28]	*n* = 28, SAH, prospective	Neurocardiogenic injury	Admission	6 min	Frequency LF, HF	500 Hz	
Swor et al., 2019 [29]	*n* = 248, ICH, prospective	Secondary brain injury	<24 h	10 s	Time SDNN, RMSSD	N.A.	14 d
Schmidt et al., 2014 [30]	*n* = 236, SAH prospective	Secondary brain injury	<48 h	3 d	Time, Frequency, MSE SDNN, RMSSD LF, HF, LF/HF Entropy	240 Hz	5 d
Odenstedt Herges et al., 2021 [31]	*n* = 55, SAH, Prospective	Secondary brain injury	<1 h	10 d	Time SDNN	1000 Hz	10 d or discharge
Wennenberg et al., 2020 [32]	*n* = 55, SAH, retrospective	Secondary brain injury Mortality	<24 h	10 d	Time, Frequency SDNN, RMSSD LF, HF, LF/HF	1000 Hz	1 year
Kox et al., 2012 [33]	N= 14 SAH (8), ICH (6), prospective	Secondary brain injury	<24 h	Series of 5 min (day 1,2,3,4)	Frequency LF, HF, LF/HF	4096 Hz	4 d
Chiu et al., 2012 [34]	*n* = 132, SAH, Prospective	Mortality	<0.5 h	10 min	Frequency LF, HF, LF/HF	1000 Hz	28 d
Uryga et al., 2018 [35]	*n* = 57, SAH, retrospective	Mortality	<24 h	6 d	Time, Frequency SDNN, RMSSD LF, HF, LF/HF	200 Hz	27 d
Sycora et al., 2020 [36]	*n* = 47, ICH, prospective	Mortality	<24 h	10 m	Frequency, MSE LF, HF, LF/HF Entropy	200 Hz	3 months
Park et al., 2013 [37]	*n* = 24, SAH, prospective	Mortality	N.A.	21 d	Time, Frequency SDNN, RMSSD LF, HF, LF/HF	200 Hz	21 d

SAH = Subarachnoid hemorrhage, HRV Heart rate variability, BP Blood pressure, GOS Glasgow outcome scale, NPE Neurogenic pulmonary edema, mRS modified Rankine Scale, DCI Delayed cerebral ischemia, SDNN normal-to-normal deviation of intervals measured between consecutive sinus beats, RMSSD root-mean-square of successive beat-to-beat differences, HR-SD mean value of HR with its standard deviation, HR-ARV heart rate average real variability, LF- HF low and high frequency components of the power spectral density, LF/HF ratio of low-frequency power/high-frequency power, MSE multiscale entropy.

**Table 3:** HRV analysis and functional outcome endpoint.

Study	Poor Functional Outcome	Results	Comments
Miwa et al., 2020 [19]	mRS > 4, at 90 days.	HR (mean value) adjOR = 1.31, 95% CI, 1.14–1.50 for 10 bpm increase. HR-ARV adjOR = 1.07, 95% CI, 1.01–1.3.	Increased mean HR and HR-ARV within the initial 24 h.
Cai et al., 2018 [20]	GOS < 4	HR-SD adjOR = 1.14; 95% CI, 1.02–1.29; *p* = 0.026	Overactivation of sympathetic modulation related to faster HR and higher HRV-SD.
Rass et al., 2021 [22]	mRS > 3, at 3 months	HR-SD adjOR = 1.29, 95% CI = 1.01–1.66, *p* = 0.045	HR-SD early predictor in ICH.
Szabo et al., 2018 [23]	mRS > 4	Normalized HF adjOR 1.2, 95%CI 1.01–1.4, *p* = 0.04. LF/HF adjOR 0.07, 95%CI 0.01–0.4, *p* = 0.02	Decreased autonomic modulation associated with poor outcome in ICH.
Chen et al., 2018 [21]	mRS > 2, at 3 months	Lower complexity index in ICH than control group (adjOR 1.09, 95% CI 1.00–1.19)	Non-linear complexity of HRV related to stroke severity, size of hemorrhage and function outcome in patients with ICH.

mRS modified Rankine scale, HR-SD heart rate variability standard deviation, HR-ARV heart rate average real variability, HF high frequency, LF/HF ratio of low-frequency power/high-frequency power.

**Table 4:** HRV analysis and cardiovascular complications.

Study	Cardiovascular Complications	Results	Comments
Megjhani et al., 2020 [24]	NCI Heart wall motion abnormality with ventricular dysfunction on transthoracic echocardiogram or cardiac troponin-I > 0.3 ng/m	Decreased vagal activity in NCI with respect to control. LF/HF (β 3.42, SE 0.92, *p* = 0.0002).	HRV and machine learning approach associated with NCI development in SAH.
Chen et al., 2016 [25]	NPE	Lower LF% (OR 0.933; 95% CI 0.910–0.958)) in NPE than non-NPE.	LF% associated with occurrence of NPE in SAH.
Kawahara et al., 2003 [26]	NCI ECG abnormalities (prolongation of QTc, presence of U wave, and ST depression)	LF/HF lower in the acute phase than in the chronic phase, and in the control group.	Augmentation of vagal activity in acute phase of SAH. No differences between the chronic phase and the control group.
Su et al., 2018 [27]	NPE	Increased LF/HF (2.7-fold, *p* = 0.03)	Complications in SAH associated to sympathetic overexcitation and vagal withdrawal
Svigelj et al., 1996 [28]	NCI ECG abnormalities (prolongation of QTc, presence of U wave, and ST changes)	HF increase between days 4 and 6 in SAH patients compared to the control group (*p* < 0.04). No significant differences in LF were found.	Failure of HRV to confirm sympathetic hyperactivity in SAH patients.

NCI neurocardiogenic injury, LF/HF ratio of low-frequency power/high-frequency power, LF% normalized power of heart rate variability spectral density in low frequency range.

**Table 5:** HRV analysis and secondary brain injury.

Study	Secondary Brain Injury	Results	Comments
Swor et al., 2019 [29]	Fever occurrence	Lower HRV associated with greater odds of fever occurrence (OR 0.92, 95% CI 0.87–0.97)	Early parasympathetic dysfunction (HRV) may improve ICH outcome.
Schmidt et al., 2014 [30]	DCI	LR^+^ 3.0 (2.3–3.8), LR^−^ 0.2 (0.1–0.5), PPV 42.4 (32.3–52.5), NPV 94.4 (90.7–98.2)	HRV changes reflect DCI complications in SAH.
Odenstedt Herges et al., 2021 [31]	DCI	71% of DCI cases identified by machine learning process, 57% of non- DCI identify as DCI.	Machine learning applied to HRV supports the prediction of DCI in SAH.
Wennenberg et al., 2020 [32]	DCI	LF/HF increased in DCI patients β = −0.07, 95% CI 0.01–0.12 *p* = 0.012.	No correlation of HRV parameters in the first 48 h with DCI development.
Kox et al., 2012 [33]	Inflammatory cytokines production	Higher HF% and lower LF/HF in ICH patients compared to control	No numerical data reported

DCI delayed cerebral ischemia, LR^+^ and LR^−^ positive and negative likelihood ratios, PPV positive predictive value, NPV negative predictive value, LF/HF ratio of low-frequency power/high-frequency power, HF% normalized power of heart rate variability spectral density in high frequency range.

**Table 6:** HRV analysis and mortality.

Study	Mortality	Results		Comments
Chiu et al., 2012 [34]	29% (In hospital)	LF/HF (OR 2.16; 95% C, 1.18–3.97; *p* = 0.013), LF% (OR 0.78; 95% CI 0.69–0.88; *p* < 0.001)		HRV analysis predictive of mortality
Uryga et al., 2018 [35]	25% (In hospital)	HF% (OR 0.63; 95% CI 0.467–0.867; *p* < 0.001)		HRV analysis predictive of mortality
Wennenberg et al., 2020 [32]		Dead SDNN 8.17 (8.11–9.33) RMSSD 7.98 (6.61–11.59)	Alive 27.6 (20.7–41.6) 23.5 (13.5–40.3)	SDNN, RMSSD, lower in died patients (*p* < 0.05). LF%, HF%, LF/HF not different in died patients and alive.
Sycora et al., 2020 [36]	25.5% (within 3 months)	Entropy (adjOR 0.09, 95% CI 0.1–0.8, *p* = 0.03)		Entropy analysis predictive of mortality in ICH.
Su et al., 2018 [27]	13.3% (within 1 week)	Increased LF/HF (2.7-fold, *p* = 0.03)		Mortality in SAH associated to sympathetic overexcitation and vagal withdrawal.

LF low frequency, HF high frequency, LF% normalized power of heart rate variability spectral density in low frequency range, HF% normalized power of heart rate variability spectral density in high frequency range, RMSSD root-mean-square of successive beat-to-beat differences, SDNN normal-to-normal deviation of intervals measured between consecutive sinus beats, LF/HF ratio of low-frequency power/high-frequency power.

## Data Availability

The data presented in this study are available on request from the corresponding author.
